# Platelet transfusion in patients with aneurysmal subarachnoid hemorrhage is associated with poor clinical outcome

**DOI:** 10.1038/s41598-020-57683-7

**Published:** 2020-01-21

**Authors:** R. Post, M. A. Tjerkstra, S. Middeldorp, R. Van den Berg, Y. B. W. E. M. Roos, B. A. Coert, D. Verbaan, W. P. Vandertop

**Affiliations:** 1 Department of Neurosurgery, Academic Medical Center Amsterdam, Amsterdam, The Netherlands; 2 Department of Vascular Medicine, Academic Medical Center Amsterdam, Amsterdam, The Netherlands; 3 Department of Radiology, Academic Medical Center Amsterdam, Amsterdam, The Netherlands; 4 Department of Neurology, Academic Medical Center Amsterdam, Amsterdam, The Netherlands

**Keywords:** Cerebrovascular disorders, Stroke

## Abstract

Patients with subarachnoid hemorrhage (SAH) who are using antiplatelet drugs prior to their hemorrhage, often receive platelet transfusions to reverse antiplatelet effects prior to life-saving surgical interventions. However, little is known about the effect of platelet transfusion on patient outcome in these patients. The aim of this study is to investigate the effect of platelet transfusion on clinical outcome in patients with aneurysmal SAH (aSAH) who use antiplatelet agents. Consecutive adult patients with an aSAH admitted between 2011 and 2015 to the Academic Medical Center (Amsterdam, the Netherlands) were included. Demographic characteristics and in-hospital complications were compared and clinical outcome was assessed after six months. Multivariable logistic regression analysis was performed to correct for confounding variables. A total of 364 patients with an aSAH were included. Thirty-eight (10%) patients underwent platelet transfusion during admission. Patients receiving platelet transfusion had worse clinical outcome (modified Rankin Scale score 4–6) at six months compared to patients without platelet transfusion (65% versus 32%, odds ratio 4.0, 95% confidence interval:1.9–8.1). Multivariable logistic regression analysis showed that platelet transfusion during admission was associated with unfavorable clinical outcome after six months; adjusted for age, treatment modality, modified Fisher and WFNS on admission (adjusted odds ratio 3.3, 95% confidence interval: 1.3–8.4). In this observational study, platelet transfusion was associated with poor clinical outcome at six months after correcting for confounding influences. In aSAH patients who need surgical treatment at low risk of bleeding, the indication for platelet transfusion needs careful weighing of the risk-benefit-balance.

## Introduction

Most patients with a history of thromboembolic vascular disease or ischemic stroke are prescribed antiplatelet therapy, and its use has increased over the last decade^1–3^. Although these drugs are certainly beneficial in aforementioned diseases^4^, they also increase the risk of major bleeding.

In case of intracranial hemorrhage, platelet transfusions are more frequently used to reverse the effect of antiplatelet agents over the past several years^5,6^. The rationale for platelet transfusion is to improve platelet activity in acute bleeding, thereby reducing the extent of the hemorrhage and potentially improving clinical outcome and survival^7–9^. The existing literature is controversial when evaluating the role of platelets (platelet transfusion and antiplatelet therapy) in intracerebral hemorrhage (ICH). Studies concerning this subject are either supporting^7,10^, questioning^11,12^ or even disapproving^13,14^, and vary in their observed effect on hematoma growth, clinical outcome, mortality, and rates of acute adverse events. Other more specific transfusion-related complications may also arise, such as acute lung injury, thrombosis, hemolytic transfusion reactions and transfusion-associated sepsis^15–19^.

Recently, the first randomized controlled trial (PATCH)^20^ addressing the efficacy of platelet transfusion in antiplatelet-associated ICH showed more adverse events and worse clinical outcome in patients who were randomized to platelet transfusion than in those without transfusion. In aneurysmal subarachnoid hemorrhage (aSAH) the impact of antiplatelet drugs on outcome has been investigated and found to be of no benefit towards a better outcome^21–23^. However, no studies have previously investigated the use of platelet transfusion in these patients.

The aim of the present cohort study was to assess the effect of platelet transfusion on clinical outcome in patients with aneurysmal SAH.

## Methods

### Study population

We performed a single-center cohort study of consecutive adult patients with aSAH, who were admitted between 2011 and 2015 to the Academic Medical Center (Amsterdam, the Netherlands), a tertiary referral center for patients with an aSAH in the Amsterdam Metropolitan Area with a total population of approximately 2.4 million people. They were eligible for this study if (1) SAH was confirmed by a plain CT-scan on admission, or CSF results that were considered positive for presence of bilirubin according to local criteria (2) a causative aneurysm was documented by CT-angiography (CTA) and/or digital subtraction angiography (DSA). Patients participating in the ongoing ULTRA study^24^ were excluded from this analysis. The institutional review board of Amsterdam UMC (IRB) reviewed the present study and granted a waiver of informed consent due to the retrospective nature of the study. All procedures and methods were performed in accordance with the updated guidelines and regulations.

### Data collection and definitions

The following items were collected: demographic characteristics, World Federation Neurological Scale (WFNS) score on admission in presenting hospital, antiplatelet agent use prior to admission (acetylsalicylic acid, dipyridamole or clopidogrel), modified Fisher grade of CT-scan on admission, aneurysm location, aneurysm treatment modality, in-hospital mortality, complications (recurrent bleeding, hydrocephalus, delayed cerebral ischemia, seizures, meningitis, infection and other complications), CSF drainage (i.e. external CSF drainage by ventricular or lumbar catheter), procedural complications (aneurysm rupture, intra-arterial thrombus or dissection) and clinical outcome. Additionally, data was collected from our transfusion laboratory regarding the platelet transfusions.

Clinical outcome was assessed after six months by the modified Rankin Scale (mRS), using a standardized telephone interview by trained and experienced specialized nurses. Antiplatelet use was dichotomized into present or absent. Recurrent bleeding was defined as a second (or third etc.) bleeding from the causative aneurysm after the initial bleeding, either diagnosed with plain CT-scan of the head or if there was a high clinical suspicion, such as an acute clinical deterioration combined with an abrupt increase in blood pressure, bradycardia, or the appearance of a sudden increase in production of CSF with fresh blood through ventricular drainage. Hydrocephalus was defined either by enlarged ventricles on imaging, assessed by an experienced neuroradiologist, or by increased intracranial pressure diagnosed by lumbar puncture or ventricular catheter placement. Delayed cerebral ischemia (DCI) was defined according to Vergouwen *et al*.^20^. Seizures were defined either by clinical appearance of rhythmic tonic or clonic movements for which anti-epileptic drugs were started or by electroencephalography (EEG). Infection was considered present when either a pulmonary infection and/or an urinary tract infection had occurred. Pneumonia was defined as clinical symptoms followed by a positive culture based on the microbiological analysis of sputum or a consolidation on the chest X-ray. Urinary tract infection was defined as clinical symptoms followed by a positive culture based on the microbiological analysis of urine or leukocytosis or bacteremia in the urine sediment. Meningitis was defined as clinical symptoms followed by a positive culture based on the microbiological analysis of cerebrospinal fluid. All results were reported according to the STROBE guidelines.

### Treatment and clinical management

Patients were treated according to our standardized protocol, which was mainly based on the International Guidelines of 2009, including calcium antagonists (Nimodipine 60 mg 6 times daily), hypertensive augmentation and normovolemia when DCI was clinically suspected. Ruptured aneurysms were treated as early as feasible (preferably within 24 hours after onset of the initial SAH). An external ventricular drain, or when possible an external lumbar catheter, was placed for CSF drainage in case of hydrocephalus, or if raised intracranial pressure was suspected. If patients were using anticoagulant therapy on admission and had an INR > 1.3, these effects were reversed with 4-factor prothrombin complex concentrate (Cofact®). When an intra-arterial thrombosis occurred during endovascular treatment lysis of the thrombus was attempted by intra-arterial injection of abciximab (Reopro®) followed by acetylsalicylic acid 100 milligram (mg) for three months. If endovascular treatment was only possible with stent-assisted coiling, patients were treated with once daily acetylsalicylic 100 mg acid and once daily clopidogrel 75 mg for three months.

### Platelet transfusion

Between 2011 and 2015 platelet concentrates were provided by Sanquin Dutch Blood Bank, according to national standards. Platelet concentrates had a volume of circa 350 mL and consisted of three components: pooled buffy coats, plasma and Platelet Additive Solution Type E (PAS-E). Buffy coats were separated by centrifugation from whole blood donations of five individual donors of identical ABO and Rh(D) compatible blood groups. The buffy coats were pooled and added to the residual plasma of one of the five donors. The pool was filtered, Fresenius® Compostop Flex, resulting in a leukodepleted platelet concentrate with no RCC in the end product, low leukocyte value and high platelet yield. Finally, the storage medium PAS-E was added. The ratio of plasma and PAS-E was 35:65 (35% plasma en 65% PAS-E). The minimal amount of thrombocytes is 250 × 109 and the residual amount of leukocytes is less than 5 × 106. In 90% of the platelet concentrates the amount of leukocytes is less than 1 × 106. The volume of the storage medium has been adjusted in such a way that optimal pH and presence of the so-called “swirling effect” is maintained. The added plasma contains hardly any labile clotting factors, only physiological levels of potassium, slightly elevated sodium and physiological to slightly elevated glucose levels.

When patients were using antiplatelet agent(s) at diagnosis of aSAH, these were discontinued regardless of aneurysm treatment and platelet transfusions were only administered at the discretion of the treating physician. All patients who needed a ventricular or lumbar catheter placement and who were using antiplatelet agents, received platelet transfusion directly before surgery at the discretion of the treating surgeon.

### Statistical analysis

In order to improve interpretability, the WFNS grade (good (WFNS grade 1–3) and poor (WFNS grade 4–5) neurological state), modified Fisher grade (no or thin hemorrhage (mFisher grade 0–2) and extensive thick hemorrhage (mFisher grade 3 and 4)), and mRS score (good (mRS 0–3) and poor outcome (mRS 4–6)) were dichotomized.

#### Descriptive statistics

Patients were classified into one of two groups: “platelet transfusion” or “no platelet transfusion”, based on whether they did, or did not, receive any platelet transfusion during admission. Patient characteristics, comorbidities, treatment modalities and complications were compared between the groups. Normally distributed variables, tested with the Shapiro Wilk test (>0.9 is normally distributed), were expressed as means with standard deviations (SD) and tested with the Student’s T test, and unequally distributed variables as medians with interquartile ranges (IQR 25–75%) and tested with the Mann–Whitney U test. The Chi-square or Fisher’s exact test was used to assess differences among groups wherever appropriate.

#### Modelling

We assessed the association between platelet transfusion and clinical outcome at six months using univariable and multivariable logistic regression analyses to calculate unadjusted (OR) and adjusted odds ratios (aOR) with 95% confidence intervals (CI). In these models, platelet transfusion was included as independent variable and the dichotomized mRS score was used as outcome variable. To identify relevant confounders (defined as variables that changed the crude OR by more than 10%)^25^, a bivariable analysis was performed with potentially confounding variables added separately to the model. Potential confounders were prior anti-platelet use, age, WFNS grade, modified Fisher, treatment modality, CSF drainage, history of a diabetes, history of cardiovascular disease, recurrent bleeding, delayed cerebral ischemia, hydrocephalus, systemic infection, meningitis, delirium, and procedural complications. The final multivariable model included platelet transfusion and confounders as independent variables. As sensitivity analysis, the final multivariable model with categorical variables instead of dichotomous variables was calculated, if applicable.

Statistical analyses were performed using the SPSS Statistics Software (IBM Corporation, New York, United States, version 24). P-values < 0.05 were considered significant.

### Data sharing statement

De-identified individual participant data that underlie the reported results will be made available 3 months after publication for a period of 5 years after the publication date at (Open Science Framework) https://osf.io.

## Results

**Figure 1:**
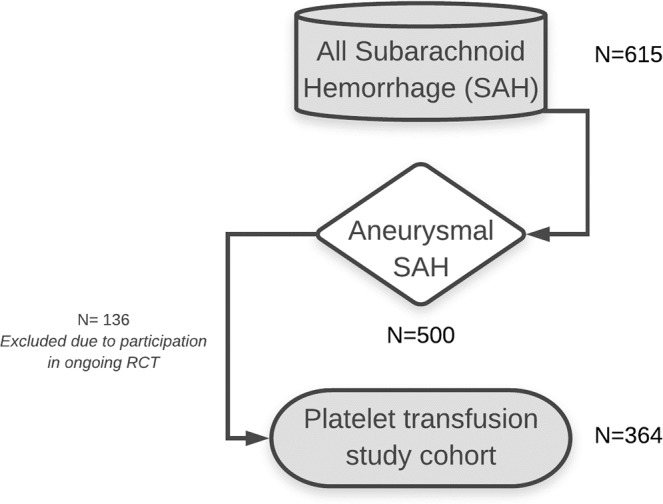
Flow-chart of selection of patients for current study.

Between 2011 and 2015, a total of 500 patients with aSAH were admitted to the Academic Medical Center (Amsterdam, the Netherlands). After exclusion of aSAH patients participating in an ongoing randomized control trial (ULTRA trial)^24^, 364 patients with aSAH were included in our study (Fig. 1). As five patients (1%) were lost to follow-up, the clinical outcome at six months was assessed in 359 patients.

The mean (SD) age of the study cohort was 57 (12) years, the proportion of women was 67% and 38% had a poor WFNS grade at time of admission. Thirty-eight (10%) patients used antiplatelet therapy prior to admission (Table 1). Patients who underwent platelet transfusion were significantly older, had a higher WFNS grade and modified Fisher score on admission, more diabetes mellitus and cardiovascular comorbidity and more frequently used antiplatelet therapy prior to admission than patients who had not undergone platelet transfusion.

**Table 1:** Baseline characteristics of 364 patients with aSAH.

	All N = 364	Platelet Transfusion N = 38	No Platelet Transfusion N = 326	p-values
Age, mean [SD]	57 [12]	63 [12]	57 [12]	0.002
Female	244 (67)	26 (68)	218 (67)	0.847
WFNS grade on admission^*^ > 3	125 (38)	21 (64)	104 (35)	0.001
mFisher scale on admission^**^ ≥ 3	324 (89)	38 (100)	286 (88)	0.024
**Aneurysm Location**^**#**^				0.886
Anterior circulation	226 (62)	24 (64)	202 (62)	
Posterior circulation	138 (38)	14 (37)	124 (38)	
**Aneurysm treatment modality**				0.023
None	57 (16)	9 (24)	48 (15)	
Coiling (+/−Stent)	244 (67)	18 (47)	226 (69)	
Clipping	63 (17)	11 (29)	52 (16)	
Antiplatelet use on admission	44 (12)	17 (45)	27 (8)	0.000
**Comorbidities**				
Cardiovascular	65 (19)	13 (36)	52 (17)	0.005
Diabetes Mellitus	22 (7)	7 (20)	15 (5)	0.001
Hypertension	120 (36)	15 (44)	105 (35)	0.287
Hypercholesterolaemia	57 (17)	8 (24)	49 (17)	0.313

N (%) unless otherwise stated, *Missing data (9.8%), **Missing data (0.3%), ^#^Missing data (2.4%).

Of 44 patients who used antiplatelet agents prior to admission, 17 (45%) received one or more platelet transfusions. Twenty-one (55%) patients, who didn’t use antiplatelet agents prior to admission, received one or more platelet transfusions (Table 2). Over the time period the frequency of transfusion was not statistically different.

**Table 2:** Characteristics of 38 patients with aSAH who received platelet transfusion during admission.

	Antiplatelet use prior to admission	
	No	Yes
Dialysis	1	0
Extensive blood loss (during surgery)	9	0
Reversal of antiplatelet effect because diagnosis SAH	3^*^	3
Severe thrombocytopenia	3	0
Surgical procedure (high risk) e.g. decompressive craniotomy, surgical aneurysm treatment (clipping), evacuation of subdural/intracranial hematoma	2^$^	6
Surgical procedure (low risk) e.q. external ventricular drainage/external lumbar drainage	3^#^	8

*Two patients received acetylsalicylic acid 500 mg intravenously after admission to hospital due to a suspicion of myocardial infarction. After the correct diagnosis of aSAH these patients underwent platelet transfusion. One patient underwent platelet transfusion because of a recurrent bleeding diclofenac was given and an external ventricular catheter was planned. ^$^One patient was started on antiplatelet therapy (acetylsalicylic acid once daily 100 mg) due to intra-arterial thrombosis during endovascular treatment. One patient used diclofenac and therefor underwent platelet transfusion before surgery. ^#^Three patients were started on antiplatelet therapy (acetylsalicylic acid once daily 100 mg) due to intra-arterial thrombosis during endovascular treatment and/or luxation of coil into normal circulation.

### Complications

Hydrocephalus occurred in 25 (66%) patients in the platelet transfusion group and in 173 (53%) patients in patients who had not undergone platelet transfusion, which was not significantly different. CSF drainage was significantly more frequently (66%) needed in the platelet transfusion group than in patients who did not undergo a platelet transfusion (46%) (OR 2.3, 95% CI: 1.1–4.6). Infections occurred significantly more often (32%) in the platelet transfusion group than in patients who did not undergo a platelet transfusion (17%) (OR 2.2, 95% CI: 1.1–4.7). All patients developed an infection in the period after they had had a platelet transfusion. Two patients presented with an aspiration pneumonia on admission which resolved with antibiotics, also both developed a new infection after platelet transfusion.

Furthermore, there were significantly more (68%) other complications in the platelet transfusion group than in the group who did not undergo a platelet transfusion (47%) (OR 2.5, 95% CI: 1.2–5.1) (Table 3).

**Table 3:** Complications during admission in 364 patients with aSAH.

	Total *N* = 364	Platelet Transfusion *N* = 38	No Platelet Transfusion *N* = 326	OR (95% C.I.)
Recurrent bleeding	73 (20)	11 (29)	62 (19)	1.7 (0.8–3.7)
Hydrocephalus	198 (54)	25 (66)	173 (53)	1.7 (0.8–3.4)
CSF drainage	175 (48)	25 (66)	150 (46)	2.3 (1.1–4.6)
Delayed Cerebral Ischemia	102 (28)	11 (29)	91 (28)	1.1 (0.5–2.2)
Seizures	44 (12)	8 (21)	36 (11)	2.1 (0.9–5.0)
Infection (urinary or pulmonary	68 (18)	12 (32)	56 (17)	2.2 (1.1–4.7)
Meningitis	14 (4)	1 (3)	13 (5)	0.7 (0.9–5.3)
Delirium	54 (15)	9 (24)	46 (14)	1.9 (0.9–4.3)
Other complications	177 (49)	26 (68)	151 (47)	2.5 (1.2–5.1)
Procedural complication	44 (16)	7 (32)	37 (15)	2.7 (1.0–6.9)
Rupture of aneurysm^*^	15 (6)	2 (9)	13 (5)	1.8 (0.4–9.0)
Intra-arterial thrombus	28 (10)	5 (23)	23 (9)	2.9 (1.0–8.5)
Dissection of vessel wall	1 (0)	0 (0)	1 (0)	0.9 (0.9–1.0)

^*^During coiling or clipping. *N* (%) unless otherwise stated.

In this cohort, overall procedural complications during aneurysm treatment occurred in 16%. Significantly more (32%) procedural complications occurred in the platelet transfusion group than in the group who did not undergo a platelet transfusion (15%) (OR 2.7, CI 95%: 1.0–6.9) (Table 3).

### Clinical outcome

**Figure 2:**
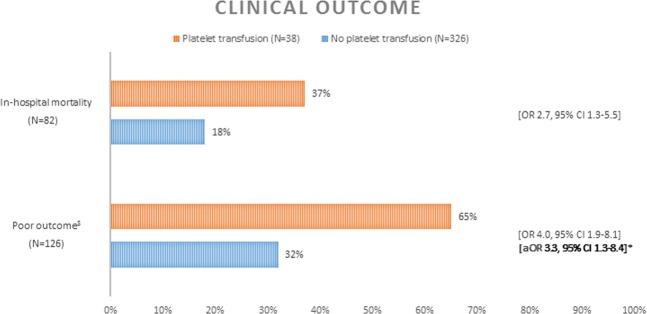
Clinical outcome in 364 patients with aSAH. ^$^Modified Rankin Scale score 4–6 at six months. *Patients included in analysis N = 244. Adjusted for age, treatment modality, modified Fisher and WFNS.

In-hospital mortality was higher in the platelet transfusion group than in the group that did not undergo a platelet transfusion (37% and 18%, respectively; OR 2.7, 95% CI: 1.3–5.5). Poor clinical outcome at six months was higher in patients who underwent platelet transfusion compared to patients who did not (65% and 32%, respectively; OR 4.0, 95% CI: 1.9–8.1). In multivariate analysis, adjusting for age, treatment modality, modified Fisher score and WFNS grade, this remained significant (aOR 3.3, 95% CI: 1.3–8.4) (Fig. 2). A sensitivity analysis, with WFNS score and modified Fisher included as categorical variables, showed also a significant association (aOR 3.7, 95% CI: 1.4–9.8) between clinical outcome and platelet transfusion.

## Discussion

In this study we noted an association between platelet transfusion and clinical outcome at six months in aneurysmal SAH patients. After adjusting for age, treatment modality, modified Fisher score and WFNS grade on admission, we found that patients who had received a platelet transfusion had a threefold increased odds to have died or to be dependent (poor clinical outcome) at six months follow-up. Furthermore, patients who underwent platelet transfusion developed more infections and this finding warrants further study.

Controversies exist regarding prior antiplatelet use^23,26^ and/or whether endovascular coiling should be followed by antiplatelet therapy in improving clinical outcome^22,27^. In a recent survey among, mostly European, neurosurgeons^28^ four percent of the responders transfuse platelets as soon at diagnosis of aSAH in patients who use prior antiplatelet therapy. In our study six patients (16%) underwent platelet transfusion after diagnosis of SAH. Evidence-based guidelines concerning (discontinuing of) antiplatelet use and SAH are lacking.

Our findings must be seen mainly as hypothesis-generating and warrant further study. Because studies regarding transfusion of platelets in aSAH are lacking, no comparison with existing literature could be made. However, some studies concerning patients with spontaneous primary intracerebral hemorrhages (ICH) did aim to describe the effect of platelet transfusion, although results of these studies are inconsistent^7,10,13,14^. Naidech *et al*. showed that platelet transfusion within 12 hours from symptom onset is associated with improved functional outcome after three months due to smaller hemorrhage sizes. These beneficial results were supported by a study by Suzuki *et al*., who showed a survival benefit of platelet transfusion in ICH patients with prior anti-platelet agents use. Creutzfeldt *et al*. found that platelet transfusion in ICH-patients did not prevent death, nor improved outcome. Also, a study by Ducruet *et al*. suggests that platelet administration does not reduce hematoma expansion in ICH patients with pre-ictus antiplatelet drugs. Nonetheless, the above-mentioned studies were all retrospective cohort studies and, according to a systematic review by Leong *et al*., the evidence for platelet transfusion in antiplatelet-related ICH was inconclusive due to methodological limitations of the included studies^11^. Based on the scarce conflicting literature an American guideline regarding the reversal of antithrombotics in intracranial hemorrhage recommends to discontinue antiplatelet agents when ICH is present and advises against platelet transfusion in patients who (1) will not undergo surgical treatment, (2) have proven platelet function within normal limits, (3) are antiplatelet resistant and (4) have used pre-ICH NSAID or glycoprotein IIb/IIIa antiplatelet treatment. They do suggest platelet transfusion in patients who have used pre-ICH aspirin or ADP-inhibitor antiplatelet therapy^29^. Recently, the first randomized controlled trial (PATCH) addressing the potential efficacy of platelet transfusion in antiplatelet associated ICH patients was performed and did show a higher death or dependency rate at three months in patients receiving additional platelet transfusion, when compared to the standard care group^20^. Furthermore, Baharoglu *et al*. found that serious adverse events were more common in the platelet transfusion group. Platelets have pro-inflammatory effects and transfusions might enhance vascular permeability associated with inflammation and platelet consumption, and although no additional explanatory mechanisms were found the authors conclude that platelet transfusion is potentially hazardous in ICH and application should only occur when supported by robust evidence^20^. Regularly, additional cytokine accumulation takes place during storage time of platelet products, resulting in increased pro-inflammatory effects, which could imply a more complex inflammatory pathophysiology in these aSAH patients. Consequently, platelet transfusion might lead to poor outcome. It may be plausible that one or more of these properties, acting alone or as a combined effect lead to poor outcome in our population. but this observed association warrants further study.

This study has some limitations. Firstly, one of the limitation of this study is the small number of patients, which makes it difficult to regain reliable estimates in extensive models, and therefore limits the analyses that can be performed. Secondly, although the findings of our study are in concordance with the PATCH-study, whether there is a true relationship between platelet transfusion and patient outcome or whether platelet transfusion reflects only the severity of a worse patient group (i.e. prior antiplatelet use, higher age and more cardiovascular history) remains uncertain. In our study the transfusion group was small, patients were older and more patients in the transfusion group had a history with cardiovascular or diabetes mellitus disease, however we corrected for confounders by logistic regression. Thirdly, our study, with its retrospective nature, limits the validity of the results and also did not systematically record adverse platelet transfusion reactions, but our prospective SAH registry however, does contain accurate data on all non-neurological complications. Perhaps mild transfusion and allergic reactions were not noted as such, but it is highly unlikely that sepsis and severe anaphylactic episodes, which are known to negatively influence clinical outcome, would have been missed. Fourthly, due to the small sample size, mortality could not be adjusted for differences at baseline. Finally, several possible important variables were not recorded including concurrent red blood cell and/or fresh frozen plasma transfusion, platelet count and platelet concentrate storage time. Some of these variables might have influenced the platelet transfusion group to a worse outcome, and therefore, our data should be interpreted with some caution and preferably be taken into consideration in future (randomized) studies. One major strength of this study is that collected data had very few missing data and 99% of clinical outcome at follow-up was completed.

In conclusion, our results show that platelet transfusion was associated with more complications during admission, higher in-hospital mortality, and poor clinical outcome after six months. Based on these results, which are in accordance with the recent PATCH trial in ICH, the indication for platelet transfusion in aSAH patients, who need surgical intervention at low risk of bleeding, needs careful weighing of the risk-benefit balance.
